# Effect of post-treatment process of microalgal hydrolysate on bioethanol production

**DOI:** 10.1038/s41598-020-73816-4

**Published:** 2020-10-07

**Authors:** Gyeongho Seon, Hee Su Kim, Jun Muk Cho, Minsik Kim, Won-Kun Park, Yong Keun Chang

**Affiliations:** 1 Department of Chemical and Biomolecular Engineering, Korea Advanced Institute of Science and Technology (KAIST), Daejeon, 34141 Republic of Korea; 2 Daegu Center, Korea Basic Science Institute (KBSI), 80 Daehak-ro, Daegu, 41566 Republic of Korea; 3 Department of Chemistry and Energy Engineering, Sangmyung University, Seoul, 03016 Republic of Korea; 4 Advanced Biomass R&D Center, Daejeon, 34141 Republic of Korea

**Keywords:** Biofuels, Applied microbiology, Microbiology techniques

## Abstract

Microalgae accumulate abundant lipids and are a promising source for biodiesel. However, carbohydrates account for 40% of microalgal biomass, an important consideration when using them for the economically feasible production of biodiesel. In this study, different acid hydrolysis and post-treatment processing of *Chlorella* sp. ABC-001 was performed, and the effect of these different hydrolysates on bioethanol yield by *Saccharomyces cerevisiae* KL17 was evaluated. For hydrolysis using H_2_SO_4_, the neutralization using Ca(OH)_2_ led to a higher yield (0.43 g ethanol/g sugars) than NaOH (0.27 g ethanol/g sugars). Application of electrodialysis to the H_2_SO_4_ + NaOH hydrolysate increased the yield to 0.35 g ethanol/g sugars, and K^+^ supplementation further enhanced the yield to 0.41 g ethanol/g sugars. Hydrolysis using HNO_3_ led to the generation of reactive species. Neutralization using only NaOH yielded 0.02 g ethanol/g sugars, and electrodialysis provided only a slight enhancement (0.06 g ethanol/g sugars). However, lowering the levels of reactive species further increased the yield to 0.25 g ethanol/g sugars, and K^+^ supplementation increased the yield to 0.35 g ethanol/g sugars. Overall, hydrolysis using H_2_SO_4_ + Ca(OH)_2_ provided the highest ethanol yield, and the yield was almost same as from conventional medium. This research emphasizes the importance of post-treatment processing that is modified for the species or strains used for bioethanol fermentation.

## Introduction

The worldwide problem of climate change and global warming has spotlighted the need for research on the use of biomass for the production of biofuels^1,2^. Microalgal biomass has significant potential for use in biofuel production because microalgae have high biomass productivity per unit area, their growth does not compete with food crops and is carbon–neutral, and they accumulate abundant lipids^3,4^. Despite the many advantages of microalgal lipids for the production of biofuels, the economic feasibility and industrialization of microalgal biofuel production require further developments^4^.

A possible breakthrough is the conversion of the other cellular components of microalgae into valuable materials^5^. Carbohydrates are among the candidates for biorefineries. Microalgal carbohydrates mostly occur as starch and cellulose and can account for up to 40% of the dry cell weight (DCW) of microalgae^6,7^. Treatment of these carbohydrates with hydrolysis or other procedures can produce more valuable carbohydrates^4^. The oleaginous microalgae, which are considered most suitable for biofuel production, consist of 30 to 60% lipids and 30 to 50% carbohydrates. Therefore, there is a need for a strategy that uses carbohydrate residue after producing biofuels^8–10^. However, the chemical treatment of these carbohydrates to produce edible value-added carbohydrate products may also produce undesirable contaminants due to the adverse effects of organic solvents^11,12^. Therefore, utilization of these microalgal carbohydrates as a source for microbial fermentation medium has been limited to ethanol production^13,14^, although there is a need for more progress in this area.

In contrast to the lignocellulosic biomass from land plants, microalgae have no lignin (which requires harsh chemical conditions for hydrolysis) and therefore generate little or no toxic compounds during hydrolysis^9,15^. For this reason, hydrolysis of microalgal carbohydrates to produce fermentable sugars, and use of these sugars for fermentation by microorganisms to produce value-added products could be an effective approach for enhancing the economic feasibility of the microalgal biofuel industry.

Previous research has mainly used acids and enzymatic catalysts for the hydrolysis of microalgae because these methods provide high production of monosaccharides^14,16^. Although enzymatic hydrolysis can produce a higher monosaccharide yield and selectivity for cell walls and starch, it is difficult to use enzymes as an industrial process because of their high cost and slow rates of hydrolysis^14^. On the contrary, acid hydrolysis is rapid and inexpensive^17^ and is therefore most suitable for the large-scale industrial hydrolysis of carbohydrates. However, there are several important considerations when using acid hydrolysis: the low pH, the degradation of sugars, and the production of compounds that inhibit microbial growth following neutralization^13^. Thus, it is necessary to optimize the acid hydrolysis and post-hydrolysis treatments^18,19^.

The optimization procedure typically considers different types of acids, different acid concentrations, and different reaction times needed to achieve the maximum yield of fermentable sugars and the lowest yield of compounds that inhibit microbial growth. For example, in the case of *Chlorella* sp. KR-1 biomass, addition of 0.3 N HCl at 121 °C for 15 min saccharified 98.2% of carbohydrates into mono-sugars, and the yield decreased when 1 N HCl was used^20^. A high acid concentration causes oxidation of sugars due to excessive hydrolysis, and generates organic acids or 5-hydroxymethylfurfural (HMF), which can inhibit growth^9^. Moreover, hydrolysis yields can differ among algal species when using the same hydrolysis conditions^21^. Therefore, the specific methods of acid hydrolysis must be optimized for different microalgal strains or species and the microbes used for fermentation. Post-hydrolysis procedures must consider which alkali to use for neutralization and control for salt additions during hydrolysis and neutralization. Some salts are necessary for microbial cell growth, and these should be considered when selecting the acid and alkali^13^. However, excessive salts can inhibit microbial growth by generating osmotic shock or the production of chemically reactive species^22,23^. Therefore, several studies have used electrodialysis (ED) to control the salt content of hydrolysates that were subjected to acid hydrolysis^13^.

In this study, the promising microalga *Chlorella* sp. ABC-001, which was recently isolated from a thermal power plant at Youngwol, Korea, was used for hydrolysis and the production of fermentable sugars. The effects of hydrolysis using H_2_SO_4_ and HNO_3_ at different concentrations and reaction times on the production of the fermentable sugars and growth inhibitors were examined. Then, the effects of post-treatment processing (neutralization and control of salt content) with precipitation and electrodialysis (ED) on the ethanol yield of *Saccharomyces cerevisiae* KL17 grown in the different hydrolysates were investigated. The effects of major salts and supplementation were also determined.

## Results and discussion

### Chemical composition of Chlorella sp. ABC-001

The growing number of industries that produce microalga-based value-added products and biofuels has created an urgent need for methods that can efficiently manage microalgal residues^24^. Thus, the effect of using different hydrolysate residues from *Chlorella* sp. ABC-001 to promote fermentation and ethanol production by *S. cerevisiae* KL17 was examined. This recently isolated microalga was confirmed as an ideal strain for the capture of CO_2_ and the utilization of cooling water from a power generation facility, because of its excellent growth rate at high concentrations of CO_2_ (up to 10%) and salt (up to 35 g/L), and at a relatively high temperature (35 °C)^25,26^.

The chemical composition of *Chlorella* sp. ABC-001 indicated the presence of abundant lipids (39.4% DCW), making this strain a highly suitable as a source of biofuel (Table 1). Fermentable and non-fermentable carbohydrates accounted for 39.1% of DCW, protein for 13.9%, and inorganic ash for 7.4%. Analysis of the fermentable monosaccharides indicated abundant glucose (67.8%), galactose (16.9%), and mannose (9.1%), all of which can be used by *S. cerevisiae* KL17 for ethanol production^27,28^. Compared with other microalgae (Table A.1), *Chlorella* sp. ABC-001 has greater potential for use as a source of carbon biomass and biofuel because of its much higher levels of lipids and carbohydrates.

**Table 1:** Chemical composition of *Chlorella* sp. ABC-001.

Macro-molecules	% DCW
Carbohydrates	39.1 ± 0.61
Lipids	39.4 ± 2.63
Proteins	13.9 ± 0.14
Inorganic ash	7.4 ± 0.32

Monosaccharides	% DCW
Glucose	67.8 ± 4.27
Rhamnose	16.9 ± 0.13
Galactose	9.1 ± 0.23
Mannose	3.4 ± 0.32
Xylose	2.1 ± 0.18
Others	0.7 ± 0.27

Elements	% DCW
C	52.4 ± 0.13
H	7.8 ± 0.01
N	2.9 ± 0.03
S	0.3 ± 0.02
O	30.5 ± 0.07

### Dilute acid hydrolysis of *Chlorella* sp. ABC-001

Because *Chlorella* sp. ABC-001 has abundant carbohydrates, the optimal procedure for acid hydrolysis was determined. Numerous acids can effectively hydrolyze microalgal biomass, but some acids, such as hydrochloric acid and phosphoric acid, are unsuitable. Hydrochloric acid is highly toxic, volatile, corrosive, and difficult to use at an industrial-scale^29^; phosphoric acid is a triprotic acid that generally produces a low sugar yield in the hydrolysis of microalgae^13^. H_2_SO_4_ and HNO_3_ are therefore often used for the hydrolysis of microalgal biomass^6,9,30^.

**Figure 1:**
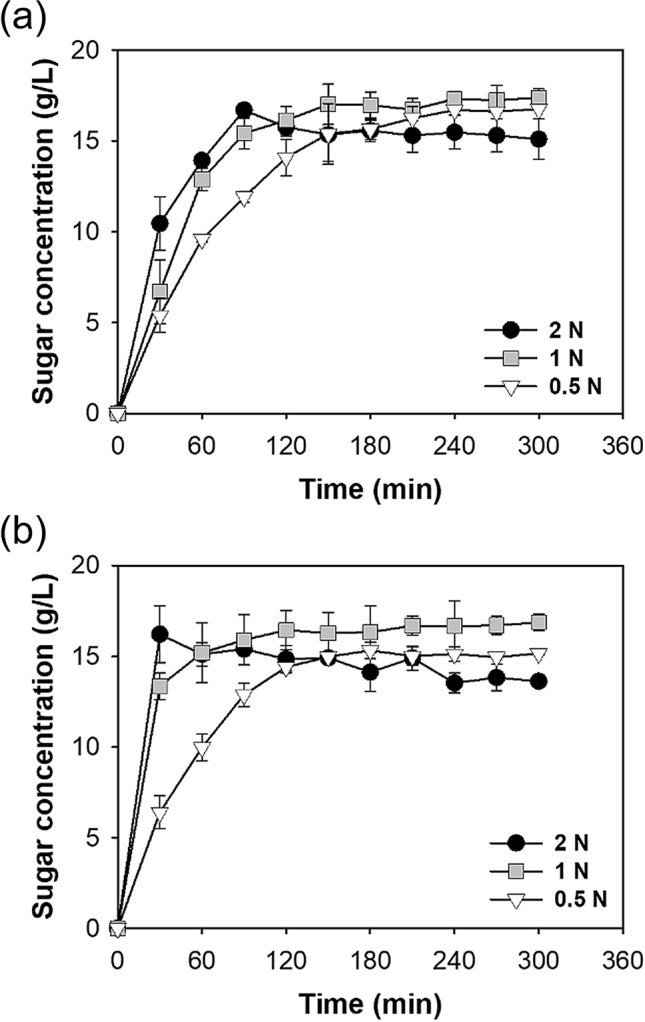
Time course of carbohydrate hydrolysis of *Chlorella* sp. ABC-001 in different concentrations of H_2_SO_4_ (**a**) HNO_3_ (**b**). ANOVA tests indicated significant differences in hydrolysis time (*P* < 0.001).

First, the effects of different acids (H_2_SO_4_ and HNO_3_), acid concentrations (0.5 to 2 N), and reaction times (0 to 300 min) on the sugar production by hydrolysis of microalgal biomass were determined (Fig. 1). For each acid, when the concentration was 2 N, the initial rate of sugar production was more rapid and the maximum was achieved more rapidly, but the sugar concentration declined slightly for long reaction times (2 N H_2_SO_4_: 16.7 g/L at 90 min and 15.1 g/L at 300 min; 2 N HNO_3_: 16.2 g/L at 30 min and 13.6 g/L at 300 min).

Use of 1 N H_2_SO_4_ led to a more gradual increase of sugar concentration to 17.0 g/L, and this level was maintained for 300 min. Similarly, use of 1 N HNO_3_ led to a gradual increase of sugar concentration to 16.4 g/L, and this level was maintained for 300 min. Use of 0.5 N H_2_SO_4_ or HNO_3_ led to slower increases of sugar concentration (16.7 and 15.1 g/L, respectively) at 300 min. Considering that a concentration of 0.5 N was not sufficient to hydrolyze all the carbohydrates and a concentration of 2 N led to a decreased amount of total sugars and the need to use additional alkali for the neutralization, a concentration of 1 N was chosen for both acids. In addition, because there were no compositional changes in the total fermentable sugars when hydrolyzed by H_2_SO_4_ or HNO_3_ (Fig. A1), a reaction time of 150 min was selected.

In all cases, there were no detectable levels of HMF or organic acids produced during acid hydrolysis, compounds known to inhibit ethanol fermentation. The decrease of sugars caused by prolonged hydrolysis at high acid concentrations could be attributable to the Maillard reaction, in which reducing sugars react with amino acids to form a brown-colored complex, a reaction known to be promoted by high acid concentrations^31,32^. Although temperature is normally considered more important than acidity in promoting this reaction^32^, the production of amino acids by protein hydrolysis can occur in the harsh conditions used for carbohydrate hydrolysis^33^.

Thus, for subsequent experiments, an acid concentration of 1 N and a hydrolysis time of 150 min was used. For H_2_SO_4_, this treatment led to a sugar concentration of 17.0 g/L (86.7% total carbohydrate) and 13.5 g/L fermentable sugars. For HNO_3_, this treatment led to a sugar concentration of 16.3 g/L (83.2% total carbohydrate) and 13.2 g/L fermentable sugars.

### Post-treatment processing of *Chlorella* sp. ABC-001 hydrolysate

Although the hydrolysates produced from H_2_SO_4_ and HNO_3_ contained abundant fermentable sugars, these hydrolysates must be neutralized before yeast cultivation because of their high acidity. Various alkalis can be used for neutralization, but these generate different salts that could adversely affect fermentation. NaOH is generally used for neutralization of microalgal hydrolysate^17,34^. Ca(OH)_2_ is also suitable following H_2_SO_4_ hydrolysis because it neutralizes the hydrolysate and the Ca^2+^ removes SO_4_^2−^ due to CaSO_4_ precipitation^18,19^. Therefore, the effects of neutralization using NaOH or Ca(OH)_2_ on subsequent yeast fermentation were examined and compared with the results in which neutralization was achieved by precipitation or ED.

A H_2_SO_4_ hydrolysate can be neutralized using Ca(OH)_2_ or NaOH. When Ca(OH)_2_ is used, CaSO_4_ precipitates and there is no need for ED. However, when NaOH is used, the H_2_SO_4_ generates Na^+^ and SO_4_^2−^ and these must be treated or removed. Although *S. cerevisiae* has a relatively high salt tolerance^23^, a growth medium with high concentrations of salts negatively impacts fermentation^13^. HNO_3_ hydrolysate does not form a precipitate when treated with Ca(OH)_2_. Therefore, only NaOH and ED were used to prepare these hydrolysates.

First, the effect of ED time on the conductivity of different hydrolysate solutions was examined (Fig. A2). Immediately after neutralization using NaOH, the conductivities of the H_2_SO_4_ and HNO_3_ hydrolysates were 57.7 and 68.0 mS/cm, respectively. ED ultimately reduced the conductivity of both hydrolysates to 5 mS/cm, the same as YPD medium. Because the SO_4_^2−^ is larger than NO_3_^−^ and close to the molecular cut-off size of the ED membrane, the time for ED processing of the H_2_SO_4_ hydrolysate was longer. The ED procedure efficiently removed Na^2+^, SO_4_^2−^, and NO_3_^−^ but did not remove sugars from the hydrolysate.

**Figure 2:**
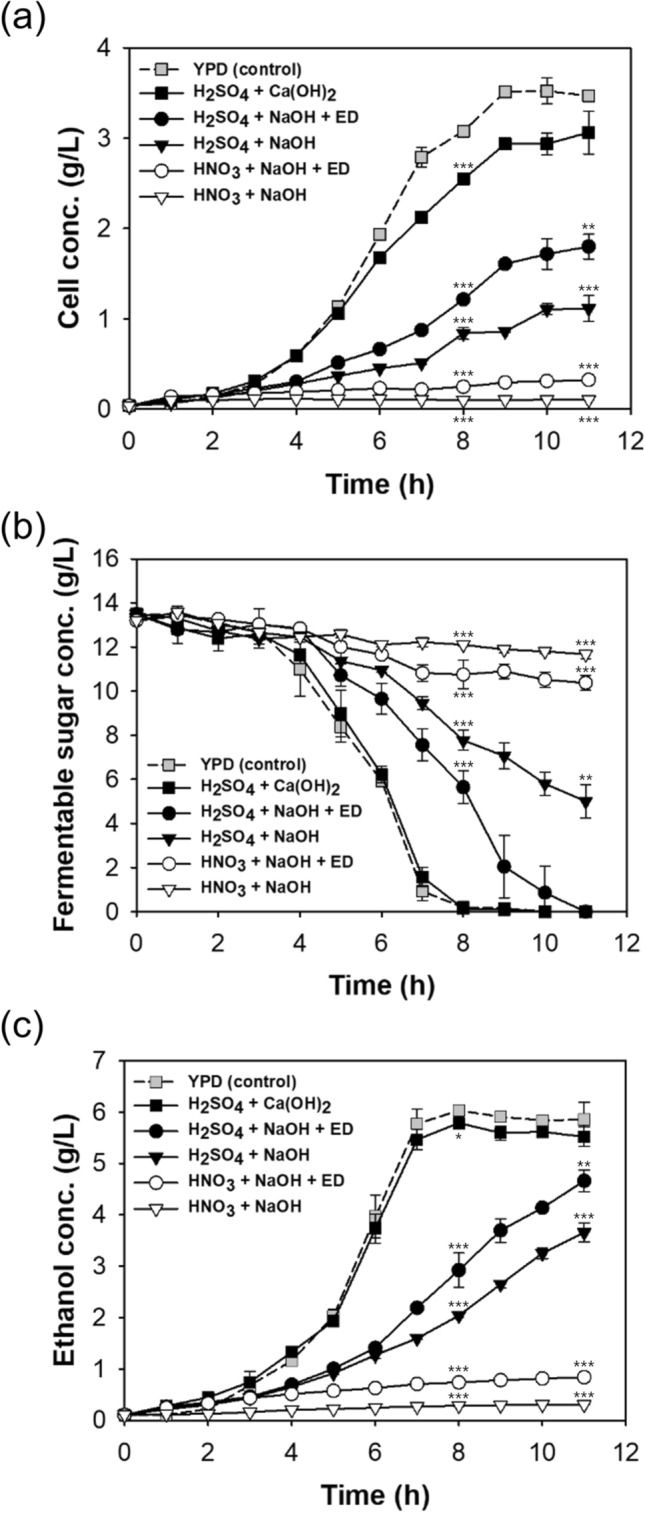
Time course of cell growth (**a**), consumption of fermentable sugars (**b**), and ethanol production (**c**) in *S. cerevisiae* KL17 using different *Chlorella* sp. ABC-001 hydrolysates or YPD. ANOVA tests at 8 h and 11 h indicated significant differences (*P* < 0.001). Student’s *t* test indicated significant differences from YPD (control), shown as ***P < 0.001, **P < 0.01, and *P < 0.05.

### Ethanol production using *Chlorella* sp. ABC-001 hydrolysate as a growth medium

#### Fermentation of *Chlorella* sp. ABC-001 hydrolysates

Next, the effect of using the different microalgal hydrolysates as substrates for bioethanol production by *S. cerevisiae* KL17 was examined. In these experiments, different hydrolysates were used as the sole culture medium and compared with YPD medium (control). *S. cerevisiae* KL17 was selected because it can ferment multiple sugars (including glucose, galactose, and mannose) that are present in the microalgal hydrolysate^27^ (Table 1).

First, cell growth, sugar consumption, and ethanol production by *S. cerevisiae* KL17 were examined using five different hydrolysates and YPD medium (Fig. 2). Most of the hydrolysates led to significantly reduced cell accumulation compared to YPD medium (3.47 g/L at 11 h, Fig. 2a). The H_2_SO_4_ + Ca(OH)_2_ hydrolysate provided the best result (3.06 g/L at 11 h), followed by the H_2_SO_4_ + NaOH hydrolysate (1.11 g/L at 11 h). The HNO_3_ + NaOH hydrolysates led to almost no growth (0.10 g/L at 11 h). In addition, ED clearly increased cell growth at 11 h (1.80 g/L for H_2_SO_4_ + NaOH + ED: 62.6% increase; 0.32 g/L for HNO_3_ + NaOH + ED: 321.6% increase). These results indicate that ED improved the fermentation of hydrolysates (presumably by removal of growth inhibitory compounds) and that the HNO_3_ hydrolysate contained growth inhibitory compounds that were not removed by ED.

Each acid hydrolysate had about 13.5 g/L of fermentable sugars, the same amount used in the YPD medium. However, the consumption of fermentable sugars differed markedly among the different treatments (Fig. 2b). The fastest consumption was in the YPD (control) medium and the H_2_SO_4_ + Ca(OH)_2_ (1.69 g/L/h) hydrolysate, and the yeast consumed all fermentable sugars by 8 h in these media. The third fastest consumption was in the H_2_SO_4_ + NaOH + ED hydrolysate, and the yeast consumed all fermentable sugars by 11 h in this medium. However, after 11 h, yeast consumed only 63.0% of sugars in the H_2_SO_4_ + NaOH hydrolysate, only 21.4% of sugars in the HNO_3_ + NaOH + ED hydrolysate, and only 11.6% of sugars in the HNO_3_ + NaOH hydrolysate. As expected, these data show that growth media that led to greater sugar consumption also led to greater cell growth.

Also as expected, the measurements of ethanol yield indicated that growth media which supported greater cell growth and sugar consumption also led to greater ethanol production (Fig. 2c). In particular, the YPD and H_2_SO_4_ + Ca(OH)_2_ groups had maximal production ethanol (6.03 and 5.78 g/L, respectively) at 8 h. At this time, all the sugars were consumed, and the ethanol yield from growth on YPD and H_2_SO_4_ + Ca(OH)_2_ hydrolysate were 0.45 and 0.43 g ethanol/g fermentable sugar, respectively. For the other growth media, the ethanol yields were markedly lower, possibly due to the presence of inhibitory factors. The most important finding of these experiments is that the H_2_SO_4_ + Ca(OH)_2_ hydrolysate can function as an effective substitute for conventional YPD medium for the fermentation of ethanol by *S. cerevisiae* KL17.

#### Effect of reactive oxygen and nitrogen species in the HNO_3_ hydrolysate of *Chlorella* sp. ABC-001

*S. cerevisiae* generally has a high salt tolerance^23^ and can be cultivated in acid hydrolysates following alkali treatment without desalting. Thus, the effects of different hydrolysates on *S. cerevisiae* fermentation were examined. The possible negative effects of reactive oxygen species, reactive nitrogen species, and cations were investigated. The cell growth experiments indicated that the H_2_SO_4_ hydrolysate led to reasonable cell growth, sugar consumption, and ethanol yield, but the HNO_3_ hydrolysate did not, even when the salts were removed by ED. Most studies of the effects of hydrolysates on cell cultures used H_2_SO_4_ as the acid catalyst^9,17–19^. During acid catalysis, HNO_3_ produces more organic acids than other acids because of its strong oxidizing effect, and these organic acids could possibly inhibit the growth of *S. cerevisiae*. However, there were no measured organic acids in the HNO_3_ hydrolysates. Therefore it was hypothesized that HNO_3_ hydrolysis might have produced reactive oxygen species (ROS) and reactive nitrogen species (RNS). These reactive species can cause exogenous oxidative and nitrosative stress to cells and even cause cell death^22^. Although *S. cerevisiae* has mechanisms that protect from these reactive species, high concentrations of ROS and RNS are lethal^35^.

**Figure 3:**
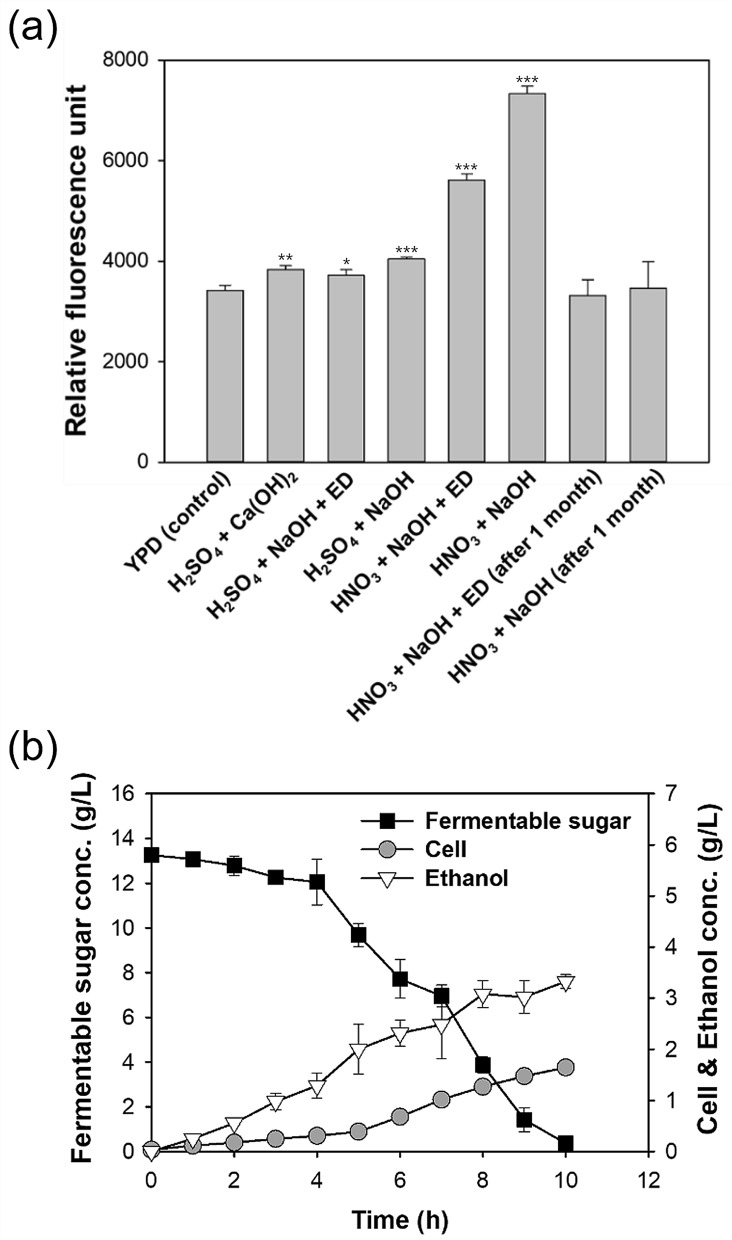
Total ROS and RNS levels (relative fluorescence units) in different freshly prepared *Chlorella* sp. ABC-001 hydrolysates and YPD, and in two hydrolysates after storage at 4 °C for 1 month (**a**) and fermentation (sugar utilization, cell growth, and ethanol production) of *S. cerevisiae* KL17 grown in 1 month-old HNO_3_ + NaOH + ED hydrolysate (**b**). ANOVA tests for (**a**) indicated significant differences (*P* < 0.001). Student’s *t* test indicated significant differences from YPD (control), shown as ***P < 0.001, **P < 0.01, and *P < 0.05.

The levels of ROS and RNS were measured using a fluorescence-based assay (Fig. 3a). All H_2_SO_4_ hydrolysates had fluorescence of approximately 4000 relative fluorescence units (RFUs) regardless of the post-treatment, and this was similar to the level in the YPD medium (3421 RFU). However, the HNO_3_ hydrolysate had high fluorescence (7333 RFU) and the level remained high (5618 RFU) even after ED. Notably, the fluorescence declined in both of these hydrolysates to about 3500 RFUs (similar to that with YPD) after storage at 4 °C for 1 month.

Thus, the effect of a HNO_3_ hydrolysate that was stored at 4 °C for 1 month on fermentation was tested (Fig. 3b). This medium led to increased cell growth (0.32 vs. 1.64 g/L), sugar consumption (0.26 vs. 1.32 g/L/h), and ethanol yield (0.06 vs. 0.25 g ethanol/g fermentable sugar; Figs. 2 and 3b). These results suggest that the oxidative stress caused by ROS and RNS was partly responsible for the inhibitory effect of HNO_3_ hydrolysate on fermentation. However, cell concentration, sugars consumption, and ethanol production were still greater in the H_2_SO_4_ + Ca(OH)_2_ hydrolysate (Fig. 2), suggesting the presence of additional inhibitory factors in the HNO_3_ hydrolysate.

#### Effects of depletion of cations in *Chlorella* sp. ABC-001 hydrolysates treated by ED

**Figure 4:**
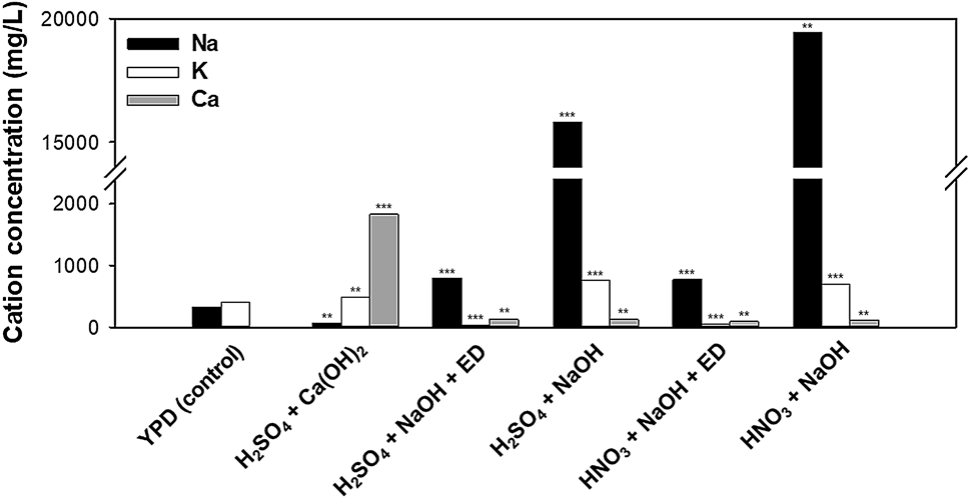
Concentrations of cations in different *Chlorella* sp. ABC-001 hydrolysates. ANOVA tests for cation concentration indicated significant differences (*P* < 0.001). Student’s *t* test indicated significant differences from YPD (control), shown as ***P < 0.001 and **P < 0.01.

The growth of *S. cerevisiae* KL17 in the HNO_3_ hydrolysate increased when the levels of ROS and RNS were reduced (Figs. 2 and 3). However, cell and ethanol concentrations remained higher in the H_2_SO_4_ + Ca(OH)_2_ hydrolysates compared with other hydrolysates that were desalted using ED (Fig. 2). This might be because ED removed certain ions that were essential for yeast growth. In particular, the molecular weight cut-off of the ED membrane allowed small ions to pass through. Thus, ICP-OES was used to measure cation concentrations of the hydrolysates after ED (Fig. 4). When NaOH was used for neutralization and desalting was not applied, the Na^+^ concentration was higher than 15,000 mg/L. The H_2_SO_4_ + Ca(OH)_2_ hydrolysate (without desalting) had a low Ca^2+^ concentration (1,825 mg/L) because of the low solubility of CaSO_4._ The K^+^ concentration in the YPD medium was 398 mg/L, and hydrolysates treated without ED had K^+^ concentrations of 450 to 750 mg/L. In contrast, hydrolysates desalted by ED had less than 40 mg/L of K^+^. K^+^ has an important role in multiple cellular processes, including regulation of intracellular pH, protein synthesis, and cell volume^36,37^. In addition, growth media with depleted K^+^ adversely affects *S. cerevisiae* growth^38^. Therefore, the removal of potassium ion by ED in these experiments could be partly responsible for inhibiting the growth of *S. cerevisiae* KL17.

**Figure 5:**
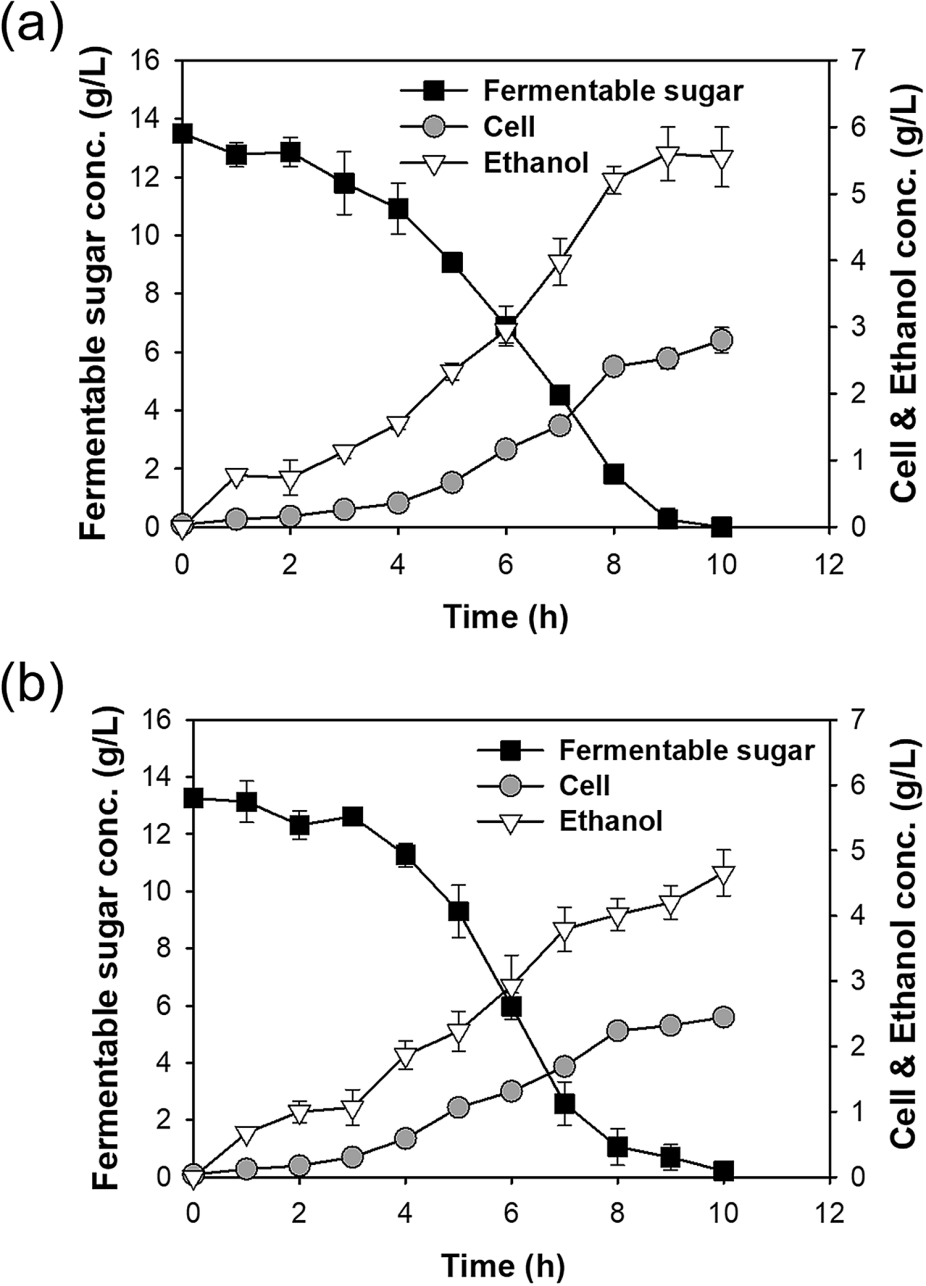
Fermentation (sugar utilization, cell growth, and ethanol production) of *Chlorella* sp. ABC-001 hydrolysate from H_2_SO_4_ + NaOH + ED + 1 g/L KCl (**a**) and hydrolysate from HNO_3_ + NaOH + ED + 1 month storage + 1 g/L KCl (**b**).

Thus, 1 g/L KCl (524 mg/L K^+^) was added to the desalted hydrolysate solutions and measured the effect on *S. cerevisiae* KL17 fermentation (Fig. 5). When the H_2_SO_4_ + NaOH + ED hydrolysate was used, the cell and ethanol concentrations at 10 h were 1.80 g/L and 4.66 g/L, respectively (Fig. 2). However, addition of 1 g/L KCl to this hydrolysate increased these values to 2.80 g/L and 5.60 g/L, respectively (Fig. 5a). These results are similar to those obtained from the H_2_SO_4_ + Ca(OH)_2_ hydrolysate (3.06 g/L and 5.78 g/L, respectively) (Fig. 2). Addition of 1 g/L KCl also improved fermentation when using the HNO_3_ hydrolysate that had reduced ROS and RNS (Fig. 5b). Therefore, the deletion of K^+^ by the desalting process appeared to adversely affect fermentation.

A comparison of the H_2_SO_4_ and HNO_3_ hydrolysates indicated that the fermentation performance depended on the alkali that was used and the post-treatment processing (Table 2). Initially, only the H_2_SO_4_ + Ca(OH)_2_ hydrolysate provided meaningful growth (3.06 g/L) and ethanol yield (0.43 g ethanol/g sugar). When NaOH as the alkali was used, the hydrolysates from H_2_SO_4_ and HNO_3_ produced remarkably decreased growth (1.11 g/L for H_2_SO_4_ + NaOH) or almost no growth (0.10 g/L for HNO_3_ + NaOH) and very little ethanol production (0.27 and 0.02 g ethanol/g sugar, respectively). This negative effect was partially reversed by the use of ED with H_2_SO_4_ (1.80 g/L and 0.35 g ethanol/g sugar), but not by use of ED with HNO_3_ (0.32 g/L and 0.06 g ethanol/g sugar). The reduction of reactive chemical species led to a partial recovery of cell growth and ethanol production (1.64 g/L and 0.25 g ethanol/g sugar).

**Table 2:** Cell growth and ethanol production by *S. cerevisiae* KL17 in different *Chlorella* sp. ABC-001 hydrolysates and YPD medium. ANOVA tests for cell growth and ethanol yield indicated significant differences (*P* < 0.001). Student’s *t* test indicated significant differences from the YPD (control), shown as ***P < 0.001, **P < 0.01, and *P < 0.05.

Acid	Neutralization	Desalting	Storage	Additive	Cell growth (g/L)	Ethanol yield (g/g sugar)
H_2_SO_4_	Ca(OH)_2_	–	–	–	3.06 ± 0.17	0.43 ± 0.01*
	NaOH	–	–	–	1.11 ± 0.10***	0.27 ± 0.01***
	NaOH	ED	–	–	1.80 ± 0.10**	0.35 ± 0.01***
	NaOH	ED	–	KCl	2.80 ± 0.13*	0.41 ± 0.02
HNO_3_	NaOH	–	–	–	0.10 ± 0.00***	0.02 ± 0.00***
	NaOH	ED	–	–	0.32 ± 0.01***	0.06 ± 0.00***
	NaOH	ED	1 month	–	1.64 ± 0.01***	0.25 ± 0.01***
	NaOH	ED	1 month	KCl	2.45 ± 0.02***	0.35 ± 0.02**
YPD medium					3.47 ± 0.01	0.45 ± 0.00

The effect of KCl supplementation on hydrolysates treated by ED was tested. KCl supplementation clearly increased the growth and ethanol production when the H_2_SO_4_ + NaOH + ED hydrolysate was used (2.80 g/L and 0.41 g ethanol/g sugar), and these values were similar to those achieved by the H_2_SO_4_ + Ca(OH)_2_ hydrolysate. However, supplementation of the HNO_3_ + NaOH + ED was less effective (2.45 g/L and 0.35 g ethanol/g sugar). Overall, the ethanol yield was always greater in the H_2_SO_4_ hydrolysates than the HNO_3_ hydrolysates, even when KCl was added (Table 2). This result suggests that additional factors in the HNO_3_ hydrolysates also inhibit fermentation. Although the effects of many post-treatments were tested, the H_2_SO_4_ + Ca(OH)_2_ hydrolysate provided optimal production of ethanol, and the results were even comparable to those from YPD medium. However, it seems likely that the optimal hydrolysis conditions and post-treatment processing will differ among different strains of microalgae and yeasts. If optimal conditions indicate the use of HNO_3_, then cost analysis and techno-economic analysis should compare the benefits from post-treatment processing, such as ED and reducing chemically reactive species.

## Conclusion

In this study, the effects of post-treatment processing of a hydrolysate from *Chlorella* sp. ABC-001 on bioethanol production by *Saccharomyces cerevisiae* KL17 grown in this hydrolysate was examined. Hydrolysates produced using H_2_SO_4_ and HNO_3_ had similar yields of sugars; however, methods used to neutralize the acids, application of ED, supplementation of K^+^, and the control of reactive species led to different bio-ethanol yields. For the microalga and yeast examined here, a hydrolysate produced by H_2_SO_4_ + Ca(OH)_2_ provided the best yield. These post-treatment processes can easily be varied, and therefore the optimization of post-treatment processing, as well as hydrolysis, is necessary.

## Materials and methods

### Cell preparation

The microalga *Chlorella* sp. ABC-001 was isolated and maintained by the Advanced Biomass R&D Center in Daejeon, Korea. The cells were stored in an incubation room (25 °C and continuous light of 120 µmol/m^2^/s) on N-8 agar plates that contained KNO_3_ (3 mM), KH_2_PO_4_ (5.44 mM), Na_2_HPO_4_ (1.83 mM), MgSO_4_·7H_2_O (0.20 mM), CaCl_2_ (0.12 mM), FeNaEDTA (0.03 mM), ZnSO_4_·7H_2_O (0.01 mM), MnCl_2_·4H_2_O (0.07 mM), CuSO_4_ (0.07 mM), Al_2_(SO_4_)_3_·18H_2_O (0.01 mM), and Bacto agar (15 g/L)^20,39^. Immediately before experiments, cells were activated in the N-8 medium with a 1 L bubble column reactor supplied with 10% (v/v) CO_2_ under 120 µmol/m^2^/s at 30 °C. To achieve sufficient biomass, cells were cultivated in a Pyrex 6 L bubble-column reactor equipped with 12 fluorescent lamps (80 µmol/m^2^/s) in N-8 medium at 30 °C. In addition, 10% (v/v) CO_2_ was supplied to the reactor at a rate of 0.75 L/min^40^. After 7 days of cultivation, cells were harvested by centrifugation at 4000 rpm for 1 min, washed with deionized (DI) water, and centrifuged again. Then, cells were lyophilized with a freeze dryer (FD8508, Il Shin Biobase Co., Korea) for 7 days, and the lyophilized cells were stored at − 20 °C prior to analysis. This process was repeated until sufficient microalgal biomass was collected. All lyophilized biomass was mixed evenly before its use in experiments.

### Composition of *Chlorella* sp. ABC-001 biomass

The total carbohydrate content of the microalgal biomass was determined using the phenol–sulfuric acid method^41^. In particular, 1 mL of a biomass solution (1 g/L) was mixed with 1 mL phenol (5% by weight), and the solution was added to 5 mL of 72% (by weight) concentrated H_2_SO_4_. The mixture was cooled to room temperature (25 °C) for 30 min in a water bath. The absorbance of each sample was measured at 490 nm using a spectrophotometer (UV-1800, Shimazu, Japan), and total carbohydrate content was calculated based on a glucose standard solution using Eq. (1) (R^2^ = 0.999):

1 $${\text{Carbohydrates}}\;{\text{concentration}}\;\left( {{\text{mg}}/{\text{L}}} \right) = 158.62 \times {\text{OD}}_{{490{\text{nm}}}} + 0.37$$

The analysis of sugar composition used the pre-treatment methods of the National Renewable Energy Laboratory^42^. First, a 50 mg sample of lyophilized cells was mixed with 0.5 mL of 72% (by weight) H_2_SO_4_ for 30 min at 30 °C, diluted to 4% (by weight) H_2_SO_4_, and then autoclaved for 20 min at 121 °C. After cooling to 25 °C, the sugars were analyzed using high performance liquid chromatography (“HPLC” section). Inorganic ash content was determined by heating in a furnace (535 °C for 3.5 h). For measurement of total lipids, 0.1 g of biomass was sonicated for 1 h with 6 mL chloroform and 3 mL methanol, and the mixture was then centrifuged at 4000 rpm for phase separation. The organic solvent phase (containing lipid) was collected and dried using N_2_ gas purging until the weight was stable, and was then measured. Protein composition was calculated based on the nitrogen-to-protein conversion factor of 4.78^43^. The elemental composition was determined using an elemental analyzer (Flash 2000, Thermo-Scientific, USA) with a thermal conductivity detector. All chemicals were purchased from Sigma-Aldrich.

### Dilute acid hydrolysis and post-treatment of *Chlorella* sp. ABC-001 solution

The acid hydrolysis of microalgae in H_2_SO_4_ (98% by weight, Sigma-Aldrich, USA) and HNO_3_ (70% by weight, Sigma-Aldrich, USA) were compared. For each test, 5 g biomass was added to 100 mL acid at different concentrations (0.5, 1, or 2 N) in an oil bath at 90 °C for 10 different reaction times (30 to 300 min). Then, the hydrolysate was cooled to 25 °C for post-treatment processing.

The post-treatment processing was used to determine the effect of chemicals generated from the hydrolyzing acids and neutralizing alkali on fermentation of ethanol by *S. cerevisiae*. For cells hydrolyzed by H_2_SO_4_, two different alkalis were compared: Ca(OH)_2_ (> 96%, Sigma-Aldrich, USA) and NaOH (> 97%, Sigma-Aldrich, USA). The CaSO_4_ was removed by sedimentation and the Na^+^ and SO_4_^2−^ ions were removed by ED. For cells hydrolyzed by HNO_3_, only NaOH was used for alkalization and Na^+^ and NO_3_^−^ ions were removed by ED.

Before the ED, the hydrolysate was passed through a membrane filter (pore diameter: 0.22 μm) for sterilization and removal of cell debris. Then, the filtered hydrolysate was desalted in the ED unit (Electro Dialyzer CJ-S3, Changjotechno Co., Korea) with an AC 110 membrane cartridge. The molecular weight (MW) cut-off size of the ED membrane was 110 (greater than the MW of SO_4_^2−^ or NO_3_^−^ and less than the MW of mono-sugars). The conductivity of the hydrolysate (an indicator of salt concentration) was measured using an electrical conductivity meter (F3, Mettler Toledo, USA). ED was continued until the conductivity of the hydrolysates was 5 mS/cm, the same as the YPD medium. Before hydrolysates were used for fermentation, they were adjusted to a pH of 5.5, and then filtered (as above) for sterilization.

### Cultivation of *S. cerevisiae* KL17

*S. cerevisiae* KL17, a strain isolated from soil in Korea^28^, was used for ethanol fermentation. For seed cultivation, cells were cultivated in YPD medium that contained glucose (20 g/L), yeast extract (10 g/L), and peptone (20 g/L). The medium used for the control group (grown in YPD medium) had a glucose concentration of 13.5 g/L. The seed culture was prepared at 30 °C and 200 rpm in a 50 mL Falcon tube containing 10 mL YPD medium. When the OD_600nm_ of the culture was 10.0, 1 mL of the seed culture medium was added to 49 mL of the main culture medium in a 250 mL flask. All main cultures were performed at 30 °C with shaking at 200 rpm. OD_600nm_ was measured and cell concentration was calculated using Eq. 2 (R^2^ = 0.995):

2 $${\text{Cell}}\;{\text{concentration}}\;\left( {{\text{g}}/{\text{L}}} \right) = 0.48 \times {\text{OD}}_{{600{\text{nm}}}} - 0.06$$

### Analysis of fermentable carbon sources, ethanol, cations, and oxidative stress

The composition and concentrations of sugars, organic acids, HMF, and ethanol in the hydrolysate were analyzed using HPLC (“HPLC” section). The concentrations of cations (Na^+^, K^+^, and Ca^2+^) in the hydrolysate were determined using an inductively coupled plasma optical emission spectrometer (ICP-OES, Spectroblue, Ametek, USA). To determine the oxidative stress of hydrolysates, total reactive oxygen species (ROS) and reactive nitrogen species (RNS) were analyzed using the OxiSelect™ in vitro ROS & RNS assay kit (STA-347, Cell Biolabs, USA) with a fluorescence microplate reader (λ_excitation_ = 480 nm, λ_emission_ = 530 nm; SpectraMax M2e, Molecular Devices)^44^.

### HPLC

Sugars, organic acids, HMF, and ethanol were measured using an HPLC system (Ultimate 3000, Dionex, USA) that had a refractive index detector and a UV detector. The temperature of the Aminex HPX-87H column was set at 65 °C and the flow rate of the mobile phase (0.01 N H_2_SO_4_) was 0.6 mL/min. The levels of galactose, xylose, and mannose were determined using an HPLC system (515 Pump, 717plus Autosampler, Waters, USA) that had an Asahipak NH2P-50 4E amine column and an evaporative light scattering detector (SEDEX 75, Sedere, France). The temperature of the column was kept at 30 °C, the mobile phase was a solution of acetonitrile and water (80:20), and the flow rate was 1.0 mL/min.

### Statistical analysis

All experiments were conducted in triplicate (n = 3) and the results were expressed as means ± SDs. A one-way analysis of variance (ANOVA) was used calculate the significance of individual differences of parameters under different conditions. Student’s *t* test was used to determine differences from the YPD (control). All calculations were performed using Microsoft Excel.

## Supplementary information

Supplementary Information.
