# Supplementary material for: Effect of post-treatment process of microalgal hydrolysate on bioethanol production
Source: Sci Rep. 2020 Oct 7;10:16698. doi: 10.1038/s41598-020-73816-4 (PMC7542428; doi:10.1038/s41598-020-73816-4)

Effect of post-treatment process of microalgal hydrolysate on bioethanol production

Gyeongho Seon[a], Hee Su Kim[b], Jun Muk Cho[a], Minsik Kim[a], Won-Kun Park[c,*], and Yong Keun Chang[a,d,*]

[a] Department of Chemical & Biomolecular Engineering, Korea Advanced Institute of Science and Technology (KAIST), Daejeon 34141, Republic of Korea

[b] Daegu Center, Korea Basic Science Institute (KBSI), 80 Daehak-ro, Daegu 41566, Republic of Korea

[c] Department of Chemistry and Energy Engineering, Sangmyung University, Seoul 03016, Republic of Korea

[d] Advanced Biomass R&D Center, Daejeon 34141, Republic of Korea

Appendix A. Supplementary data

Table A.1. Composition of microalgae used in this study and previous studies that analyzed the potential use of different microalgae for production of biodiesel.

**Microalga**	**Carbohydrates (%DCW)**	**Proteins (%DCW)**	**Lipids (%DCW)**	**Reference**
***Chlorella* sp. ABC-001**	**39.1**	**14.1**	**39.4**	**This study**
Chlorella vulgaris	21.0	41.5	15.7	Wang et al. (2013)
Nannochloropsis gaditana	25.8	43.8	18.0	Teri et al. (2014)
Spirulina platensis	11.0	42.3	11.0	Sydney et al. (2010)
Dunaliella tertiolecta	13.9	29.4	11.4	Sydney et al. (2010)

Wang, K., Brown, R. C., Homsy, S., Martinez, L., & Sidhu, S. S. (2013). Fast pyrolysis of microalgae remnants in a fluidized bed reactor for bio-oil and biochar production. *Bioresource technology*, *127*, 494-499.

Teri, G., Luo, L., & Savage, P. E. (2014). Hydrothermal treatment of protein, polysaccharide, and lipids alone and in mixtures. *Energy & fuels*, *28*(12), 7501-7509.

Sydney, E. B., Sturm, W., de Carvalho, J. C., Thomaz-Soccol, V., Larroche, C., Pandey, A., & Soccol, C. R. (2010). Potential carbon dioxide fixation by industrially important microalgae. *Bioresource technology*, *101*(15), 5892-5896.

Figure A.1. Time course of mono-sugar production of *Chlorella* sp. ABC-001 during hydrolysis by 1 N concentrations of H_2SO_4 (a) and HNO_3 (b).

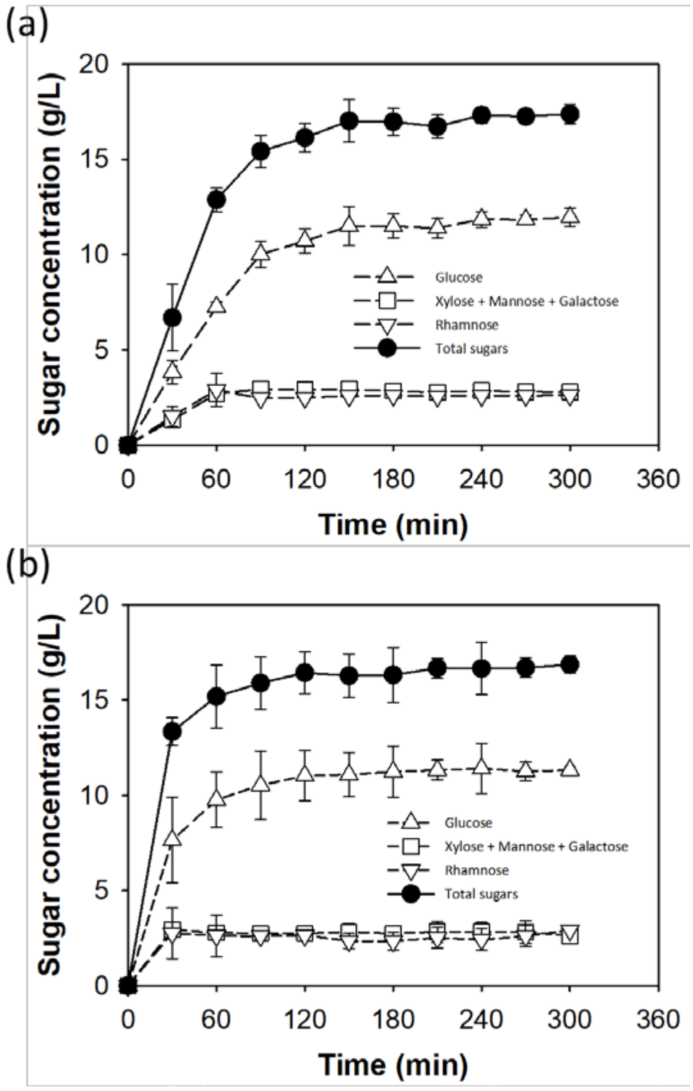

Figure A.2. Time course of changes in conductivity of two *Chlorella* sp. ABC-001 hydrolysates during electrodialysis.

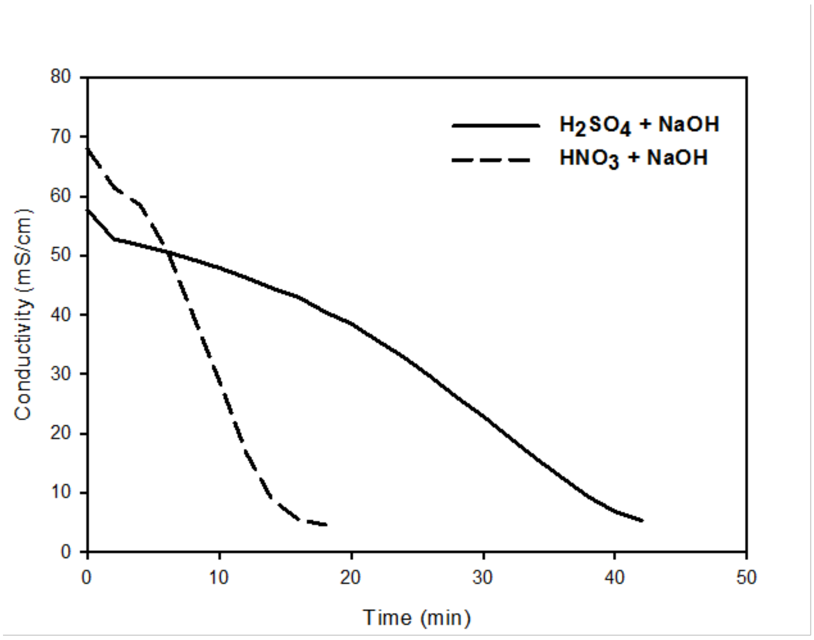

Supplement: Supplementary file 1 — Supplementary Information. [file 41598_2020_73816_MOESM1_ESM.pdf]
